# Carbadox has both temporary and lasting effects on the swine gut microbiota

**DOI:** 10.3389/fmicb.2014.00276

**Published:** 2014-06-10

**Authors:** Torey Looft, Heather K. Allen, Thomas A. Casey, David P. Alt, Thaddeus B. Stanton

**Affiliations:** United States Department of Agriculture, National Animal Disease Center, Agricultural Research ServiceAmes, IA, USA

**Keywords:** carbadox, antibiotics, microbiome, phylotype, 16S rRNA, digital PCR

## Abstract

Antibiotics are used in livestock and poultry production to treat and prevent disease as well as to promote animal growth. Carbadox is an in-feed antibiotic that is widely used in swine production to prevent dysentery and to improve feed efficiency. The goal of this study was to characterize the effects of carbadox and its withdrawal on the swine gut microbiota. Six pigs (initially 3-weeks old) received feed containing carbadox and six received unamended feed. After 3-weeks of continuous carbadox administration, all pigs were switched to a maintenance diet without carbadox. DNA was extracted from feces (*n* = 142) taken before, during, and following (6-week withdrawal) carbadox treatment. Phylotype analysis using 16S rRNA sequences showed the gradual development of the non-medicated swine gut microbiota over the 8-week study, and that the carbadox-treated pigs had significant differences in bacterial membership relative to non-medicated pigs. Enumeration of fecal *Escherichia coli* showed that a diet change concurrent with carbadox withdrawal was associated with an increase in the *E. coli* in the non-medicated pigs, suggesting that carbadox pre-treatment prevented an increase of *E. coli* populations. In-feed carbadox caused striking effects within 4 days of administration, with significant alterations in both community structure and bacterial membership, notably a large relative increase in *Prevotella* populations in medicated pigs. Digital PCR was used to show that the absolute abundance of *Prevotella* was unchanged between the medicated and non-medicated pigs despite the relative increase shown in the phylotype analysis. Carbadox therefore caused a decrease in the abundance of other gut bacteria but did not affect the absolute abundance of *Prevotella*. The pending regulation on antibiotics used in animal production underscores the importance of understanding how they modulate the microbiota and impact animal health, which will inform the search for antibiotic alternatives.

## Introduction

Antibiotics are used in animal agriculture for both therapeutic and non-therapeutic applications (Animal Health Institute, 2012). Appropriately high doses of antibiotics are administered to treat or prevent disease (therapeutic use), and relatively low doses are typically used to improve feed efficiency (non-therapeutic use). These differing doses of antibiotics are important because they are at the core of regulatory efforts, with many countries banning or regulating non-therapeutic veterinary antibiotics that have human medical importance (European Union, 2003; FDA, 2012). This is because low-dose antibiotics can result in bodily concentrations of antibiotics that are subinhibitory to bacteria. Indeed, it is likely that both therapeutic and non-therapeutic doses of antibiotics can lead to subinhibitory antibiotic concentrations for some host-associated bacteria. Subinhibitory antibiotic concentrations are undesired because they can have adverse effects, in particular enhancing the selection for antibiotic resistance genes and their horizontal transfer (Barbosa and Levy, 2000; Smith et al., 2002; Barlow, 2009; Brewer et al., 2013), thus promoting the antibiotic resistance problem in animal and human pathogens.

Carbadox is a quinoxaline-di-N-oxide antibiotic compound that is fed to almost a third of nursery-age pigs in the US to control enteric diseases and improve feed efficiency (USDA, 2007). Medicated early weaning, including carbadox, is credited with nearly eradicating the enteric pathogen *Brachyspira hyodysenteriae* in domestic swine [cause of swine dysentery (Stanton et al., 1999)]. Carbadox inhibits bacteria by intercalating DNA and causing mutations, and this mutagenic property has led to its ban in many countries (Beutin et al., 1981; Chen et al., 2009). The current US regulation includes a 42-day withdrawal period prior to slaughter to prevent carbadox residues in the carcass (Joint FAO/WHO Expert Committee on Food Additives, 2003). It is unclear if it will be further regulated in the US because carbadox is not an antibiotic of human clinical importance (FDA, 2003).

We are interested in carbadox because of its importance to the US swine industry and its unknown effects on swine gut bacteria. One specific collateral effect of carbadox is the induction of prophages or prophage-like gene transfer agents, as has been shown *in vitro* in Shiga toxin-producing *Escherichia coli* (Kohler et al., 2000), *Salmonella enterica* serovar Typhimurium (Bearson et al., 2014), and *B. hyodysenteriae* (Stanton et al., 2008). In addition to these results, research in our lab on total swine fecal phages suggested that prophages were induced in pigs that were fed either carbadox or ASP250 (penicillin, chlortetracycline, sulfamethazine) (Allen et al., 2011). Further identification of the effects of carbadox on the swine gut microbiome could lead to a greater understanding of its mechanism of growth promotion.

Here we analyzed the bacterial component of the swine fecal microbiota in samples taken prior to and during carbadox treatment, as well as periodically during the 6-week withdrawal period. We found that carbadox altered bacterial membership and community structure relative to non-medicated pigs, including a reduction in total bacteria. This study is an important step toward defining the effects of carbadox on the swine gut microbiome, which in turn will lead to informed alternatives to this antibiotic.

## Materials and methods

### Swine

Piglets were acquired and managed in accordance with the National Animal Disease Center Animal Care and Use Committee guidelines, as previously described (Allen et al., 2011). At 3 weeks of age, 12 piglets from 2 litters were divided into two rooms of six pigs each, with equal representation of littermates and gender. All pigs were fed a standard starter diet (TechStart® 17-25, Kent Feeds, Muscatine, IA) *ad libitum* for 3 weeks, after which six control pigs continued to receive non-medicated feed while the other group received feed containing carbadox (50 g/ton). After 21 days of continuous feed with or without carbadox, all pigs (60 days old) were switched to a non-medicated maintenance diet (Pork Finisher diet, Kent Feeds). The age of pigs receiving carbadox and transitioning to maintenance diet are consistent with standard industry practices.

**Figure 1:**
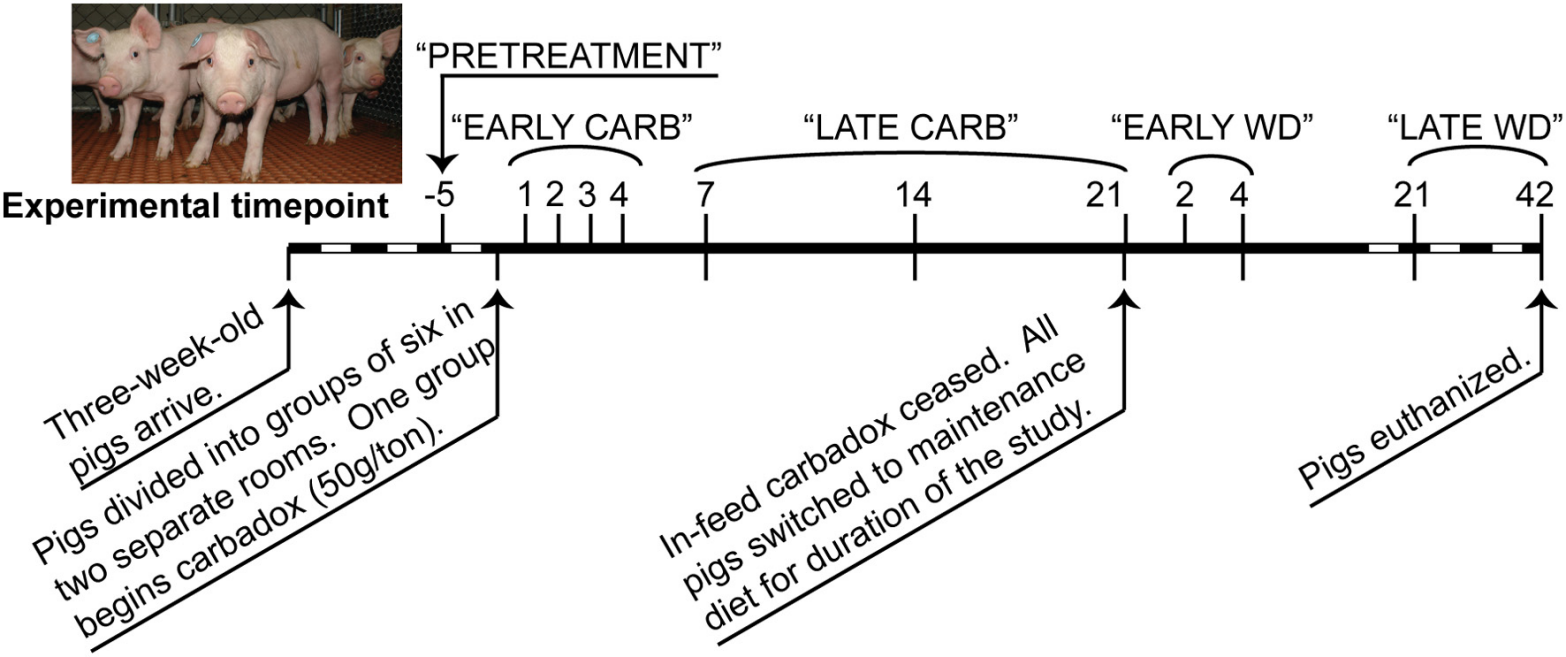
**Timeline of carbadox feed-trial**. Sample days are noted above the thick line, and dates are noted below. Dashed region not to scale. Timepoints that were grouped for some statistical analyses are labeled along the top. CARB, carbadox; WD, withdrawal.

Feces were collected from each pig at multiple times before, during, and after antibiotic withdrawal (Figure 1), and DNAs were extracted with the PowerBiome DNA Isolation Kit using the manufacturer's protocol (Mo Bio Laboratories, Solana Beach, CA, USA).

### 16S rRNA gene sequencing

Amplification of the V1-V3 region of bacterial 16S rRNA genes from individual samples was carried out as previously described (Allen et al., 2011). Primers 8F (5′-AGAGTTTGATCCTGGCTCAG) (Weisburg et al., 1991) and 518R (5′-ATTACCGCGGCTGCTGG) (Muyzer et al., 1993) were designed with an eight-nucleotide unique sequence barcodes (Hamady et al., 2008; Allen et al., 2011). PCRs were performed for 22 cycles, and the products were separated by gel electrophoresis and purified using the MinElute kit (Qiagen Inc., Valencia, CA). Amplicons were sequenced on a 454 Genome Sequencer (GS) FLX using the manufacturer's protocol for Titanium chemistry (Roche Diagnostics, Branford, CT).

### Sequence analysis

Sequence data that passed Roche's quality thresholds were processed by AmpliconNoise (Quince et al., 2011), mothur (Schloss et al., 2009, 2011), and Uchime (Edgar et al., 2011) to denoise sequencing data, remove barcodes, and reduce sequence artifacts produced during PCR. The Schloss analysis pipeline SOP was followed (Schloss et al., 2011). Briefly, mothur's implementation of the AmpliconNoise program reduced sequencing and PCR artifacts, then sequences were aligned to the Silva bacterial database (Quast et al., 2013). Screen.seqs, filter.seqs, and pre.cluster commands were performed in mothur to improve the quality of the dataset, and Uchime was used to remove chimeras (using the sequences of each sample as their own reference). OTU-based phylogenetic analysis (97% similarity cutoff) and hypothesis testing were performed with normalized data in mothur (Schloss et al., 2009). Data were normalized by subsampling each sample to 3454 reads and unless otherwise stated, and samples were analyzed by time and treatment (Figure 1). To visualize changes in community structure, the Bray-Curtis dissimilarity statistic (OTU data) and Pearson's correlations (vectors of environmental variables) were calculated and plotted by nonmetric multidimensional scaling (NMDS) in PAST (Hammer et al., 2001). Community metrics such as diversity (Shannon index, inverse Simpson index), evenness (Heips index), and richness [best parametric model in CatchAll (Bunge, 2011; Bunge et al., 2012)] were calculated in mothur based on the OTU data. Multiple *t*-tests were performed on community metrics in GraphPad Prism v. 6.02 (La Jolla, CA). Statistical significance was determined using the Holm-Sidak correction for multiple comparisons when comparing medicated vs. non-medicated at a timepoint, or using the Kruskal-Wallis test with Dunn's multiple comparison correction when comparing the all days of the carbadox group to pre-treatment (alpha = 5%). The resilience index was calculated from Shannon, Heips evenness, and inverse Simpson diversity indices using the formula described by Shade et al. (2012) based on the diversity index of the microbiota of the carbadox-treated animals at pre-treatment, at the most significantly changed time (day 7), and at the first time that was not significant from pre-treatment (day 14). Samples were analyzed by treatment per timepoint, or by treatment per range of time (pre-treatment, early carbadox exposure [days 1–4], late carbadox exposure [days 7–21], early withdrawal [days 2 and 4], and late withdrawal [days 21 and 42]; Figure 1). For most statistics, samples were compared between pigs in the medicated and non-medicated groups at each time.

Taxonomic assignments of the 16S rRNA gene sequences were made using the Ribosomal Database project (RDP) web tools, with training set version 9.0 (Cole et al., 2009). The Metastats statistical software was used in mothur for making comparisons between samples and identifying trends (White et al., 2009). Analysis of similarities (ANOSIM) was also performed in mothur to test whether difference between groups were significant.

Linear discriminant analysis effect size (LEfSe) was performed using the LEfSe web tool on taxonomic assignments from RDP's sequence classifier (Cole et al., 2009; Segata et al., 2011). The LEfSe program was used to identify indicator organisms most likely to explain the differences between treatment groups with a logarithmic cutoff value of linear discriminant analysis (LDA) > 4.0.

### Quantification of prevotella (by digpcr and qPCR)

Digital PCR (digPCR) counts the number of target molecules in each DNA sample, enabling reliable estimates of the absolute number of target molecules in the original sample (feces), without the need for an internal standard. Digital PCR was performed to estimate the number of copies of *Prevotella* 16S rRNA genes in fecal DNA from carbadox-fed and non-medicated animals at day 4 of carbadox treatment (early exposure). The Quantstudio 3D digPCR system (Life Technologies) was used according to the manufacturer's recommendations and each sample was run in triplicate. *Prevotella*-specific digPCR primers were adapted from Mieszkin et al. (2009): Bac32 Fm, 5′AACGCTAGCTACAGGCTTAAC; Bac108R, 5′ CGGGCTATTCCTGACTATGGG; Bac82Probe, 5′ **6-FAM**-ACGGGTGAG/**ZEN**/TAACGCGTATCCAAC-**IBFQ** (fluorophore and quenchers in bold). All reactions were performed using QuantStudio 3D Digital PCR Master Mix (Life Technologies, Carlsbad, California) following manufacturer's recommended cycling conditions. Each 15 μL reaction consisted of 1.0 μM of each primer, 0.2 μM probe, 1.0 ng or 0.1 ng DNA. *Prevotella* cells per gram of feces were calculated using the DNA quantity extracted from 0.5 grams of feces, and assuming a *Prevotella* spp. average of two 16S rRNA gene copies per cell [average of 58 *Prevotella* genomes available on the Integrated Microbial Genomes (IMG) website (Markowitz et al., 2012)].

Quantitative PCR (qPCR) was performed on the same samples as digPCR to evaluate the relative amount of *Prevotella* to total 16S rRNA gene copies (all bacteria). *Prevotella*-specific primers were used (F, 5′CGGGTTGTAAACTGCTTTTATGAAG; and R, 5′CGCTCCCTTTAAACCCAATAAA) as previously described (Okabe et al., 2007). Universal bacterial primers targeting the 16S rRNA gene (341F, 5′ CCTACGGGRSGCAGCAG; and 529R, 5′ ACCGCGGCKGCTGGC) (Baker et al., 2003) were used to amplify all bacterial 16S rRNA genes. All reactions were done using iTaq Universal SYBR Green Supermix following manufacturer's recommended conditions (Bio-Rad, Hercules, California). Each 20 μL reaction consisted of 0.01 ng fecal DNA and the *Prevotella* or universal primers at 0.2 μM or 0.5 μM, respectively. Relative quantification was calculated using the Pfaffl method (Pfaffl, 2001) and *t*-tests were performed in PAST (Hammer et al., 2001).

### *E. coli* viable cell populations

One gram of fresh feces was suspended in 10 ml LB broth. Samples were vortexed vigorously to make a slurry for 10-fold serial dilutions in phosphate buffered saline. Duplicate dilutions were plated on MacConkey agar medium (March and Ratnam, 1986) and incubated at 37°C. Lactose positive colonies (fecal coliforms, predominantly *E. coli*) were enumerated on the countable dilution the following day.

### Data presentation

A *P*-value less than 0.05 with a *q*-value (false discovery rate) less than 0.05 was considered significant, and *R* between 0 and 0.3 was considered a slight correlation while *R* greater than 0.3 was considered a correlation. Data are deposited in NCBI's Short Read Archive (SRA) under accession numbers SAMN02645017-SAMN02645066 and are associated with BioProject PRJNA237795.

## Results

### Carbadox alters bacterial membership early in the treatment

**Figure 2:**
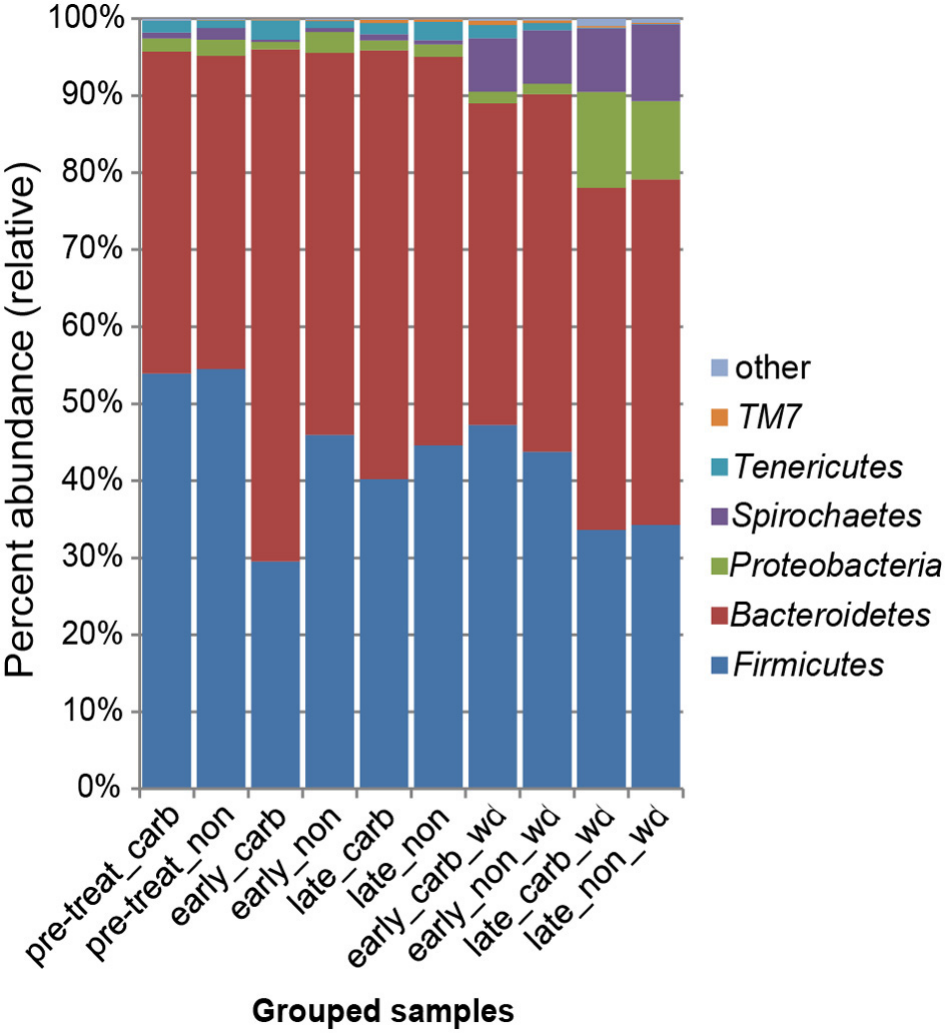
**Bacterial phyla representation in the microbiota, based on taxonomic inference of bacteria (16S rRNA sequences)**. Data are pooled by treatment and period of time. Carb, carbadox; wd, withdrawal; non, non-medicated.

We first examined differences in bacterial membership at the phylum and genus taxonomic levels during the first week of carbadox treatment. The results showed that the *Firmicutes, Proteobacteria, Elusimicrobia, Planctomycetes*, and *Lentisphaerae* phyla were of lower relative abundance, while the *Bacteroidetes* phylum was of higher abundance in the medicated animals (*q* < 0.03) (Figure 2). Interestingly, *Bacteroidetes* populations increased in the medicated animals proportionately to the decrease in *Firmicutes* populations (~17% change). This early carbadox-mediated transition to a *Bacteroidetes-*dominant microbiota was also seen when comparing early medicated animals to pre-treatment (~25%). Genus-level taxonomic assignments were also analyzed, revealing significant differences between the medicated and non-medicated animal microbiotas during the early carbadox time points. Many genera showed a relative decrease with antibiotic treatment (Figure S1), most of which belonged to the *Firmicutes* phylum (*q* < 0.05). The relative increase in *Prevotella* spp. was reflected in the increase in *Bacteroidetes* in the carbadox-treated animals (*q* < 0.05).

**Figure 3:**
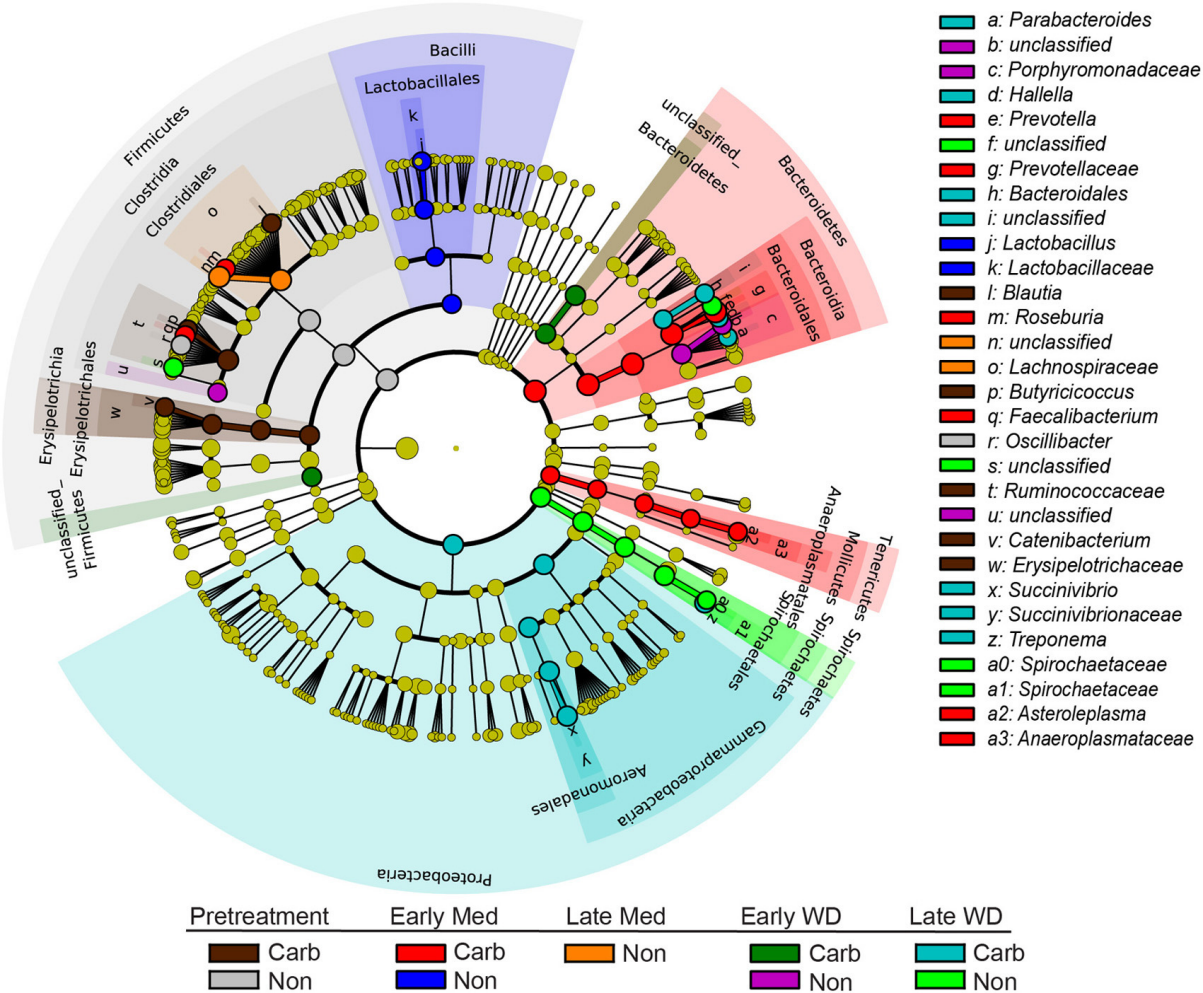
**Taxonomic tree showing biomarkers that were significantly enriched with either antibiotic exposure or lack of exposure over time**. The LEfSe-generated tree shows bacterial nodes (diameter is relative to abundance in community) with a taxonomic hierarchy. Samples were grouped together as either carbadox-treated (Carb) or non-medicated (Non-med) over the period of the study as pre-treatment, early exposure, late exposure, early withdrawal, late withdrawal. No biomarkers were identified for the late carbadox samples. The cutoff value of linear discriminant analysis (LDA) > 4.0.

The LEfSe analysis was used to resolve bacterial taxa associated with antibiotic treatment, which could define biomarkers for carbadox effects. This analysis explains why biological samples differ using tests of consistency and effect size estimation (Segata et al., 2011), with the results suggesting members of the community that benefit from or contribute to the community-wide effects described above. The results showed four genera to be enriched in early samples from carbadox-fed animals: *Prevotella, Roseburia, Faecalibacterium*, and *Asteroleplasma* (Figure 3 and Figure S2). *Lactobacillus* was enriched in samples from the early non-medicated animals (Figure 3 and Figure S2). This indicates that a few key members of the community could be the drivers of community dynamics.

### Carbadox causes rapid changes in bacterial community structure

**Figure 4:**
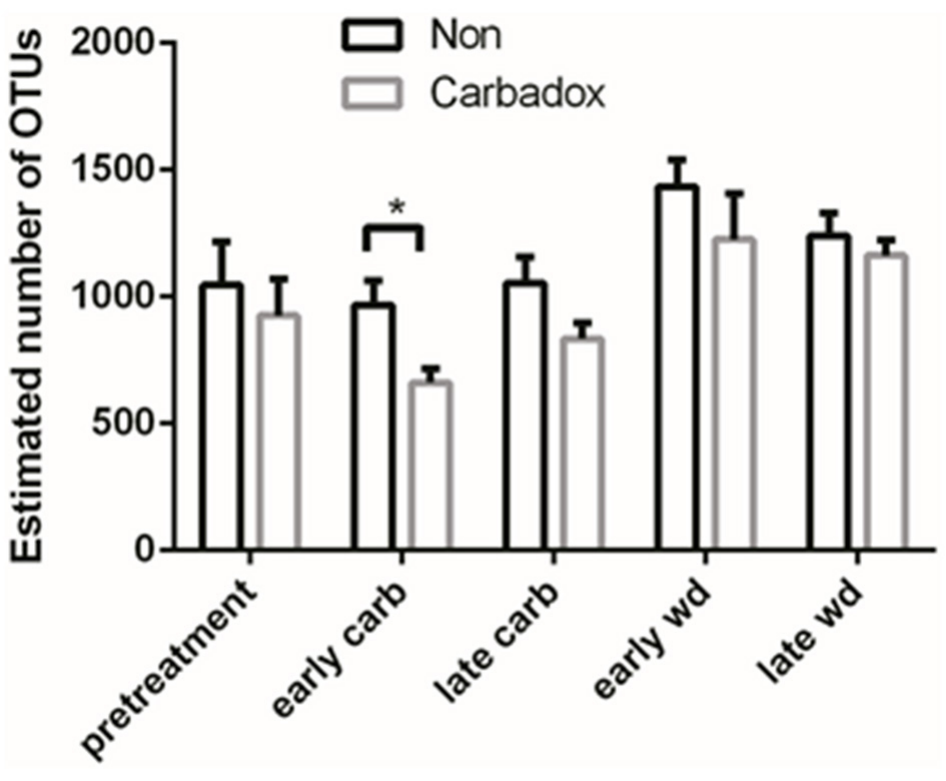
**Operational Taxonomic Unit (OTU) estimates, averaged by treatment and time**. Bars indicate the standard error around the mean. Multiple comparisons star indicates significant difference at timepoint.

**Figure 5:**
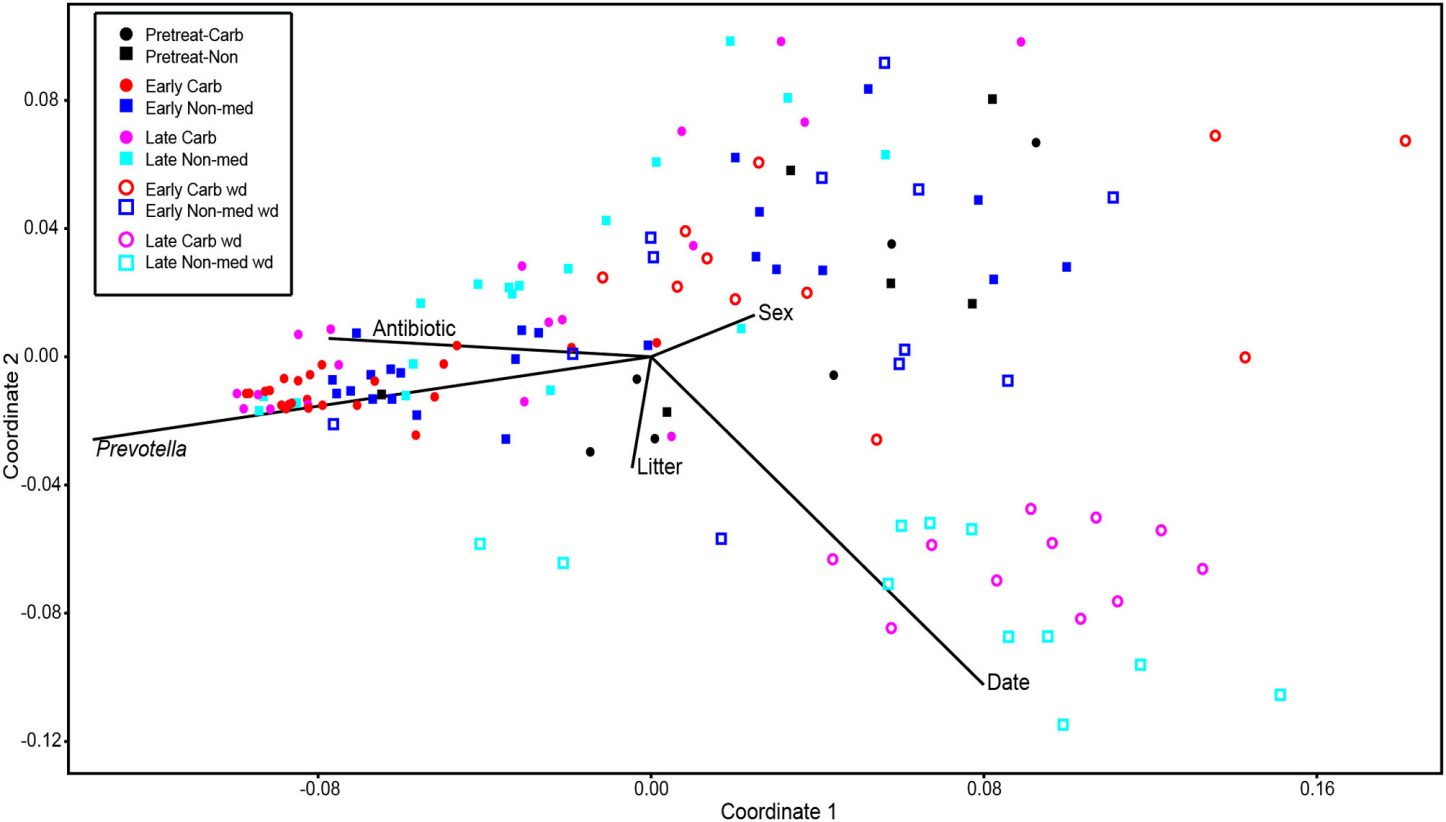
**Non-metric multidimensional scaling analysis of OTU-based bacterial 16S rRNA gene sequence abundances in individual pig samples**. Environmental variables are plotted as vectors. The length of each vector is arbitrarily scaled, so only their directions and relative lengths should be considered. Stress proportion = 0.1656.

Comparisons of community structures apply broad measures of similarities among bacterial communities in their entirety, allowing inferences to be made about populations as a whole. The effects of dietary changes on bacterial community structure were first analyzed via OTUs binned at the 97% similarity level. Estimates of the total number of OTUs (bacterial richness) during the early treatment period were significantly lower for the communities in medicated animals (661 ± 55 vs. 962 ± 96, respectively; Figure 4). Additional measures of alpha diversity (Shannon diversity, Heips evenness, and inverse Simpson indices) of samples from medicated animals compared to non-medicated animals were significantly different at 2, 3, and 4 days after continuous carbadox, but not different in either late carbadox or at any time during the withdrawal period (Figure S3). Analysis of the community structure yielded further support, showing significant differences at days 3 and 4 of early carbadox treatment ([*R* = 0.32, *p* = 0.015] and [*R* = 0.54, *p* = 0.003], respectively), but not before starting antibiotic treatment (*p* = 0.82; Figure 5). Communities from medicated animals at the remaining time points did not clearly separate from those of the non-medicated animals. The alpha diversity indices of the microbiota from the carbadox-treated animals at each sample time were compared to their pre-treatment values to determine when the microbiota recovered. Significant differences were revealed from the pre-treatment diversity at days 2, 3, 4, and 7 but at no other times, including the withdrawal period (*p* < 0.01). The swine gut microbiota showed similar resilience in response to carbadox disturbance regardless of the diversity index used to calculate it (0.16, 0.17, 0.19 from the Heips evenness, inverse Simpson, and Shannon diversity indices, respectively). Together these data show that carbadox caused an initial decrease in both bacterial richness and evenness, and that the swine gut bacterial community structure recovered after 1 week of carbadox initiation despite the continued presence of carbadox.

### Comparison of absolute and relative differences in microbiota membership

Because of the altered microbial diversity and reduction of richness early in the antibiotic treatment, digital PCR was used to evaluate whether statistically significant changes in the bacterial community of medicated animals was due to absolute changes in bacterial membership or due to reductions of carbadox-sensitive organisms. The relative abundance of *Prevotella* was most significantly impacted by carbadox and so was chosen for analysis by digPCR to determine if this was an absolute or relative change in the *Prevotella* population. Analysis of early carbadox samples (day 4) showed no significant differences in *Prevotella* counts (LOG_10_) per gram of feces between medicated (10.0, *SE* = 0.11) and non-medicated animals (9.8, *SE* = 0.26). This suggests that observed differences in *Prevotella* sequence data are due to decreases of other bacterial species rather than to increases in absolute numbers of *Prevotella.* To verify the results of the phylotype analysis and to compare with the digPCR results, qPCR was performed on the same day 4 samples. Quantitative PCR of *Prevotella* abundances, relative to total 16S rRNA genes, confirmed a 1.8-fold increase in the relative abundance of *Prevotella* in the carbadox-fed animals (*p* < 0.05). These data demonstrate the value of using digPCR to resolve the absolute abundance of taxa that show relative changes in high-throughput phylotype analyses.

### Carbadox abrogates *E. coli* population shifts during a dietary change

Culturing bacteria directly from feces also yields absolute counts, but selecting individual groups requires *a priori* knowledge and available selective media. Because carbadox is often used to prevent enteric diseases in young swine, we cultured *E. coli* from fecal samples at different experimental time points to monitor this species for potential enteric pathogens. No significant differences in *E. coli* colony forming units (CFUs) were observed during the carbadox-treatment portion of the study or late in the withdrawal period (Figure S4). Interestingly, *E. coli* CFUs were significantly different between the medicated and non-medicated groups on day 2 after the withdrawal of carbadox, with the difference being driven by increased *E. coli* CFUs in samples from non-medicated animals at that time (Figure S4). The diet for all animals was switched (consistent with industry practice) concomitant with the withdrawal of carbadox (see Methods), and this result suggests that the change in diet caused a brief increase in *E. coli* populations that was prevented in the pigs previously fed carbadox.

### Carbadox modifies the development of the swine gut microbiota

This study involved a time series that encompassed 21 days of antibiotic treatment and 42 days following its withdrawal, enabling the examination of the development of the swine gut microbiota. Members of the *Firmicutes* phylum were relatively more abundant at the beginning of the study (54% of total community) than at the end of the withdrawal period (34%) (*q* < 0.01; Figure 2). An increase in the relative abundance of other phyla [*Proteobacteria, Spirochaetes, Planctomycetes, Fibrobacteres, Synergistetes* (*q* < 0.01)] was observed over the 9-week study. Much of this maturation happened late in the experiment regardless of antibiotic treatment. However, carbadox treatment did exert some significant changes on the microbiota even after its withdrawal. Analysis of community structure revealed significant differences between the medicated and non-medicated groups late in the withdrawal period (21 days [*R* = 0.49, *p* = 0.001] and 42 days [*R* = 0.54, *p* = 0.001]), but not in the early withdrawal period (days 1, 2, 3, or 4) (Figure 5). This suggests significant changes in bacterial membership over time because there were no significant differences in comparisons of community metrics, such as richness and diversity, during the withdrawal period (Figure 4 and Figure S3). The LEfSe analysis suggested bacterial members were relatively increased with these late-withdrawal differences, including *Succinivibrio, Hallella*, and *Treponema* in the late-withdrawal medicated animals and *Spirochaetaceae* in the late non-medicated animals (Figure 4). Taken together, these results show that the swine gut bacterial community changes over time, and that carbadox influences these microbiotas even several weeks after its removal.

## Discussion

Here we define short- and long-term effects of in-feed carbadox and its withdrawal on the swine intestinal microbiota. Carbadox is one of the most common antibiotics used in the US swine industry, with indications for both disease prevention and feed-efficiency improvement. We are interested in defining the effects of carbadox to inform both its mechanism-of-action and non-antibiotic alternatives (with similar effects on production performance). Carbadox was administered continuously for 3 weeks, constituting an ecological press rather than pulse disturbance to the microbiota (Shade et al., 2012). The results show that carbadox immediately and significantly altered the bacterial community, but it did not show the same effects from 1 to 3 weeks of continual administration. This demonstrates that the swine gut microbiota was initially disturbed by carbadox, but the microbial community structure recovered despite the continued presence of carbadox. Interestingly, the discontinuation of carbadox resulted in enduring effects at the species level but not on community metrics such as diversity, suggesting that carbadox altered the membership but not the structure of the community. Similar dynamics were observed in a study of a human undergoing press β-lactam therapy, which showed an immediate reduction in *Firmicutes* populations after 6 days, a reduction in overall species richness, and subsequent reestablishment of *Firmicutes* populations after 2 weeks (Perez-Cobas et al., 2013). Furthermore, analysis of physiochemical and microbial variables in a batch reactor has shown that microbial communities can enter alternative stable states that have consequences on ecosystem processes (Burgmann et al., 2011). Further work is needed to determine how the carbadox-altered microbiota interacts with the host and how this relates to feed efficiency.

The most dramatic bacterial change was the relative increase in the *Prevotella* population during the first 4 days of carbadox exposure. However, based on the digPCR results, this was not due to an absolute increase in the *Prevotella* population, which was unchanged compared with samples from non-treated pigs, but rather to a reduction in other members of the microbiota. *Prevotella* is a well-studied swine commensal bacterium and has been identified as one of the most abundant genera in the pig intestine (Leser et al., 2002; Lamendella et al., 2011; Looft et al., 2012). The relative number of intestinal *Prevotella* has been shown to decrease after amoxicillin exposure (Mozes et al., 2013), suggesting that the antimicrobial effect on this genus is specific to the antibiotic being administered. *Prevotella* spp. metabolize recalcitrant food, such as hemicelluloses and pectin in the swine intestinal tract, which is important to animal health because fermentation end products, from these substrates, supply the host with a large portion of its energy supply. *Prevotella* was identified with *Roseburia* and *Faecalibacterium* as biomarkers that increase in relative abundance soon after carbadox administration. These bacteria are metabolically complementary since *Prevotella* produces acetate, and *Roseburia* and *Faecalibacterium* consume acetate to produce butyrate (Duncan et al., 2004a,b). Butyrate is a short chain fatty acid (SCFA) that has been shown to benefit host health (Flint et al., 2012; Furusawa et al., 2013). Additionally, links between bacterial fermentation products and host energy regulation suggest bacterial roles for improved feed efficiency. A recent study of fructo-oligosaccharides and other soluble fibers showed an intestinal microbiota shift in mice (increased *Bacteroidetes* populations and decreased *Firmicutes*) that resulted in increased SCFA production, specifically acetate, propionate, and butyrate (De Vadder et al., 2014). This led to increased concentrations of propionate in the blood, which induced intestinal gluconeogenesis to benefit glucose and energy homeostasis (De Vadder et al., 2014). Although the relative *Prevotella* increase in response to carbadox was relatively short-lived, and not an absolute change, studies of food-producing animals suggest that small health advantages at key production stages (e.g., weaning, transport, etc.) can convey significant performance improvements over time and at slaughter (Alexopoulos et al., 2004).

The health benefits of microbial SCFA production has been well documented, and certain diets and antibiotic alternatives have been shown to be particularly good modulators of SCFAs. Interestingly, studies on the host health- and microbiota-modulating effects of some prebiotics, such as dietary fiber, have shown microbiota shifts similar to what was shown in the present study with carbadox. The prebiotics arabinoxylan and inulin similarly caused a relative increase in *Prevotella, Roseburia*, and *Faecalibacterium* populations, thereby increasing propionate and butyrate production (Ramirez-Farias et al., 2009; Scott et al., 2014). *Prevotella* spp. also had relative increases after soluble fiber and non-starch polysaccharides were added to pig feed, and this diet was also associated with increased abundance of butyryl-coenzyme A (CoA) CoA transferase gene copies in feces (Metzler-Zebeli et al., 2010). It is as yet unclear if these microbiota shifts are related to the improved feed efficiency observed with agricultural antibiotics such as carbadox, but further studies are warranted.

One unexpected discovery was that carbadox abrogates a potential bloom in *E. coli* populations as a result of a diet change. Previous results from our lab and others have suggested that increased *E. coli* populations are a collateral effect of some ecosystem disturbances, including antibiotics (Janczyk et al., 2007; Looft and Allen, 2012). An abrupt change in diet has been shown to increase *E. coli* O157:H7 shedding in sheep (Kudva et al., 1997) and in pigs, diet change and weaning is associated with increase susceptibility to enterotoxigenic *E. coli* infection, a leading cause of post-weaning diarrhea (Wu et al., 2007). In the present study, we found that the initial carbadox disturbance caused no such *E. coli* increase. However, due to the pigs advancing age over the course of our extended experiment, the diet was changed from a standard nursery diet to a grower/maintenance diet (consistent with industry practice) at the same time that carbadox was withdrawn. This dietary change caused an increase in *E. coli* populations in the non-medicated animals, but not in the pigs that had previously been fed carbadox, demonstrating a protective effect of prior carbadox administration.

In contrast to the potential modulation of SCFA production described above, other commonly accepted mechanisms of how in-feed antibiotics improve feed efficiency are related to bacterial disease suppression, the reduction of the host's microbial load, or both (Dibner and Richards, 2005; Mathew et al., 2007). Our study showed a significant reduction of bacterial species richness after 4 days of continuous carbadox administration, confirming that the bacterial load is reduced albeit temporarily. Reduced bacterial richness has also been observed with the use of other in-feed antibiotics such as ASP250 (chlortetracycline, sulfamethazine, and penicillin) in swine (Allen et al., 2011). Regarding the potential mechanism of disease suppression, an intriguing result from our study is that the family *Spirochaetaceae* was lower in the carbadox-treated animals than the matched non-medicated animals 42 days after the withdrawal of carbadox. The *Spirochaetaceae* family includes *Brachyspira hyodysenteriae*, which is the causative agent of swine dysentery and one of the primary reasons carbadox is used in pig production in the US (Stanton et al., 1999). As efficacious alternatives to in-feed antibiotics continue to be explored, these data are a reminder of the need to diversify the antibiotic alternative armament with specific tools of targeting pathogens, such as vaccines, in addition to feed additives that are general modulators of bacterial community structure (Allen et al., 2013).

### Conflict of interest statement

The authors declare that the research was conducted in the absence of any commercial or financial relationships that could be construed as a potential conflict of interest.
