# Supplementary material for: Carbadox has both temporary and lasting effects on the swine gut microbiota
Source: Front Microbiol. 2014 Jun 10;5:276. doi: 10.3389/fmicb.2014.00276 (PMC4050737; doi:10.3389/fmicb.2014.00276)
Supplement: Supplementary file 1 [file DataSheet1.PDF]

Supplementary Material

Carbadox has both temporary and lasting effects on the swine gut microbiota

Looft T.[1*#], Allen H.K.[1*#], Casey T.A.[1], Alt D.P.[1], Stanton T.B.[1]

[1] National Animal Disease Center, Agricultural Research Service, United States Department of Agriculture, Ames, IA, USA

[#] Contributed equally

*** Correspondence:**
T Looft, Food Safety and Enteric Pathogens Research Unit, National Animal Disease Center, Agricultural Research Service, United States Department of Agriculture, 1920 Dayton Ave, Ames, IA 50010, USA. E-mail: torey.looft@ars.usda.gov

HK Allen, Food Safety and Enteric Pathogens Research Unit, National Animal Disease Center, Agricultural Research Service, United States Department of Agriculture, 1920 Dayton Ave, Ames, IA 50010, USA. E-mail: heather.allen@ars.usda.gov

1. Supplementary Figures

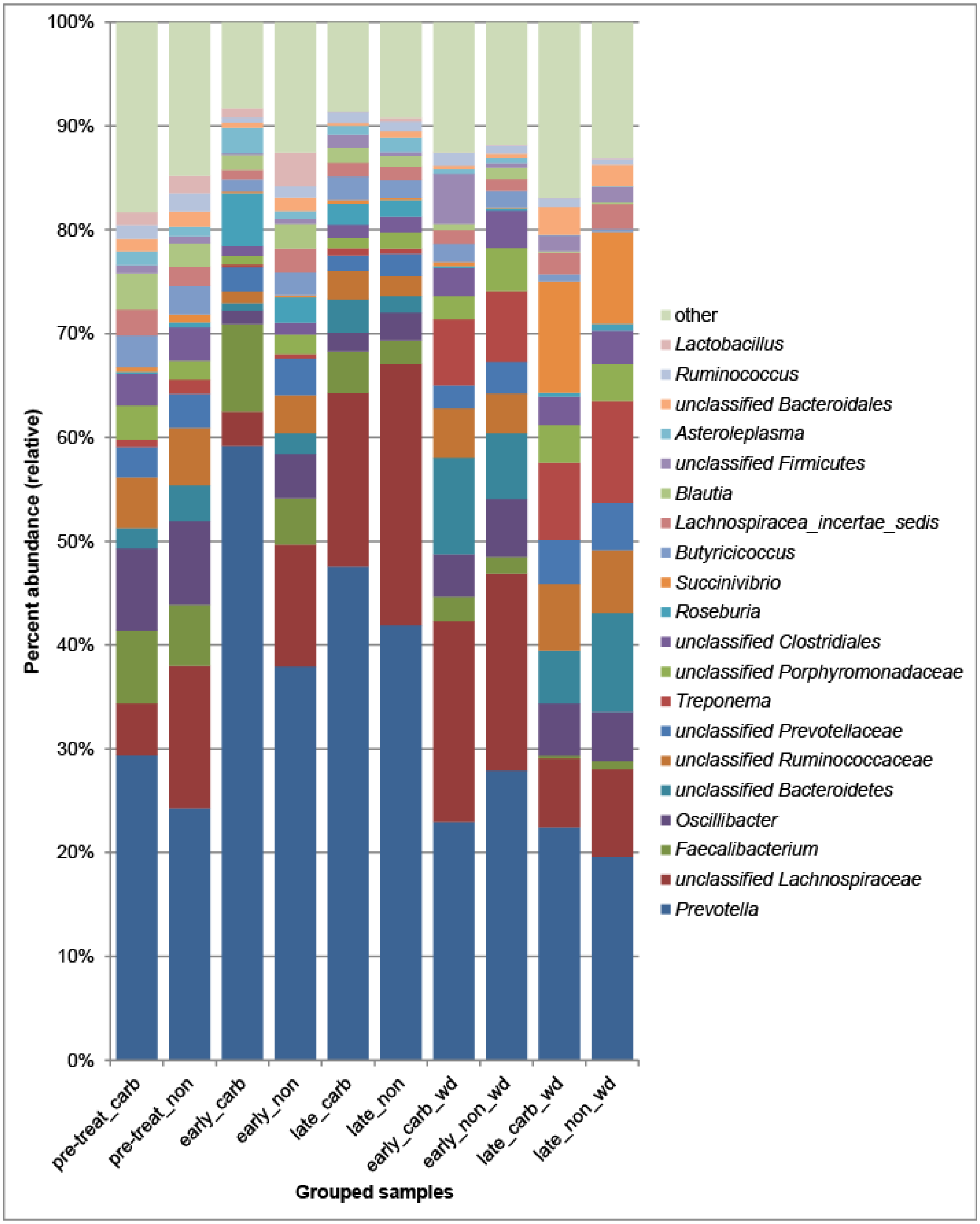

Supplementary Figure 1. Bacterial genera present in the microbiota, based on taxonomic inference of bacteria (16S rRNA sequences). Data are pooled by treatment and period of time.

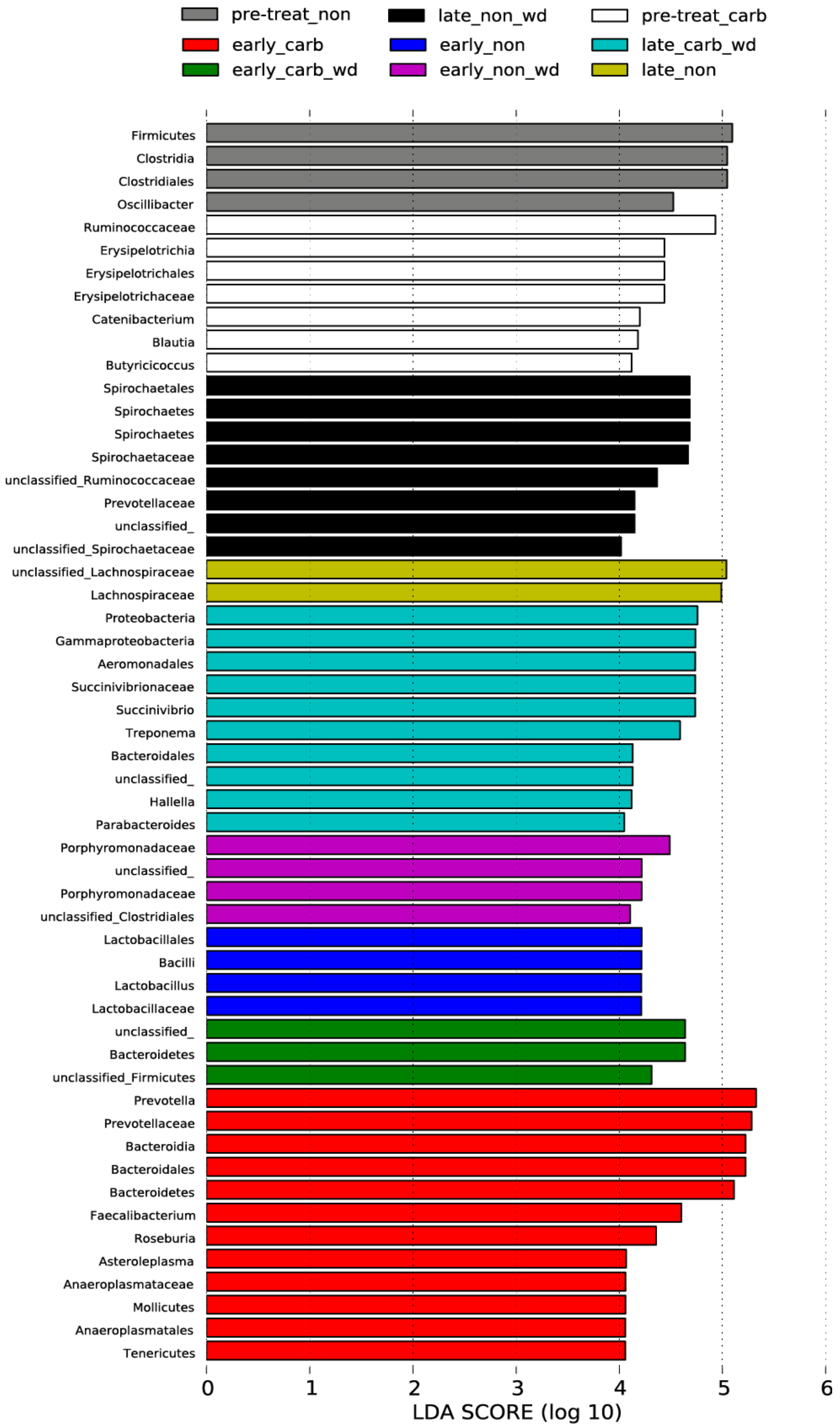

Supplemental Figure 2. Biomarkers that were significantly enriched with either antibiotic exposure or lack of exposure over time, identified with LEfSe web tool. Logarithmic cutoff value of the linear discriminant analysis (LDA) > 4.0; *p*<0.01

31

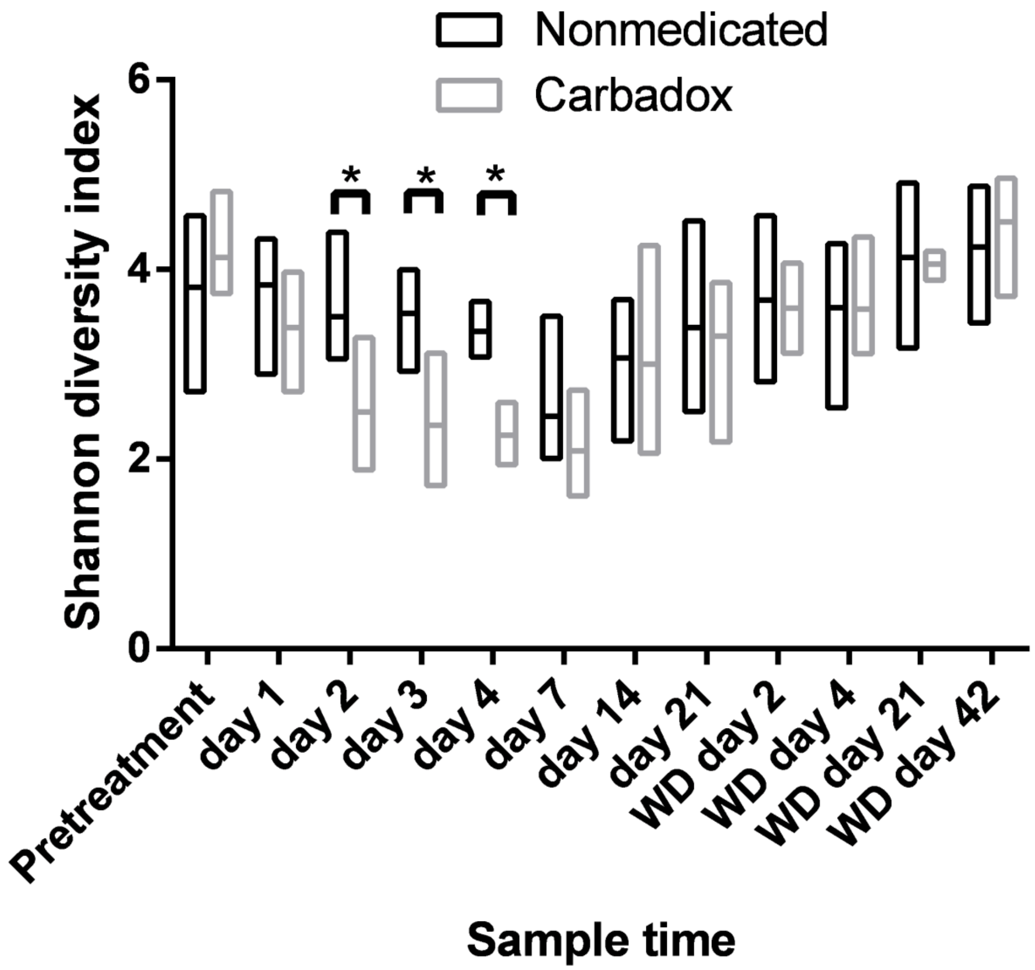

32

33 **Supplementary Figure 3.** Shannon diversity indices per sample time. The range (box) is shown
34 around the mean (bar). Significant comparisons (Holm-Sidak correction on multiple t-tests, $p<0.01$)
35 are noted with an asterisk.

36

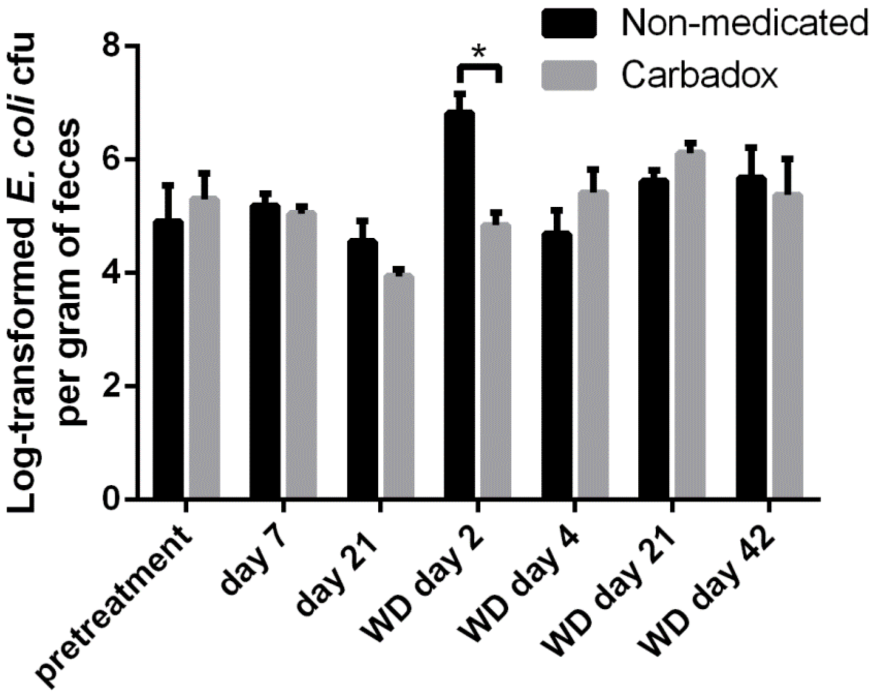

Supplemental figure 4. Prior carbadox treatment prevents and increase in *E. coli* populations caused by diet change. *E. coli* were cultured on MacConkey medium, and lactose-positive cfu were enumerated in duplicate. The average of the duplicate counts were log-transformed and plotted with the standard error around the mean of six biological replicates. Asterisk denotes statistical significance after correcting for multiple pairwise comparisons ($p<0.01$).
